# Complete Cervical Avulsion with Intravaginal Misoprostol for Second Trimester Pregnancy Termination

**DOI:** 10.1155/2012/931497

**Published:** 2012-08-02

**Authors:** G. R. Sajjan, Neelamma Patil, Manpreet Kaur, Shobha Shirgur, Suvarna Nandi, V. Ashwini

**Affiliations:** ^1^Department of Obstetrics & Gynecology, BLDE University Shri B. M. Patil Medical College Hospital and Research Centre, Bijapur 586103, Karnataka, India; ^2^Bijapur Liberal District Educational University's Shri B. M. Patil's Medical College Hospital and Research Center, Bijapur 586103, Karnataka, India

## Abstract

Intravaginal misoprostol, a synthetic PGE1 analogue, has largely replaced all other techniques for pregnancy termination in II trimester, because of its successful results. Incidence of II trimester pregnancy termination has also increased in the present days, because of prenatal diagnosis of pregnancies with serious fetal abnormalities like cardiovascular and skeletal malformations. But there are serious and life threatening complications reported with the use of intravaginal misoprostol. Here we are reporting a case of complete avulsion of cervix from lower part of the uterus, with the use of intravaginal misoprostol, for II trimester termination of pregnancy. So, clinicians dealing with II trimester termination of pregnancy should be aware of such complications.

## 1. Introduction

 Misoprostol, a synthetic PGE1 analogue, has replaced all other techniques for pregnancy termination, especially in II trimester, because of its excellent success rate [1]. World over midtrimester abortions constitute only 10%–15% of all induced abortions, but they are responsible for two-thirds of all major complications [2]. Though there are few retrospective studies showing that there is no increased risk of uterine rupture in women undergoing second trimester termination of pregnancy with misoprostol [3], there are many case reports of serious catastrophic complications like uterine rupture and postabortal bleeding necessitating hysterectomy and blood transfusions with the use of intravaginal misoprostol [4]. These are reported in both scarred uterus as well in unscarred uterus [4].

 In this paper, we are describing a case of a completely avulsed cervix from lower uterine segment in an unscarred uterus, in a woman undergoing second trimester pregnancy termination with intravaginal misoprostol.

## 2. Case Report

**Figure 1:**
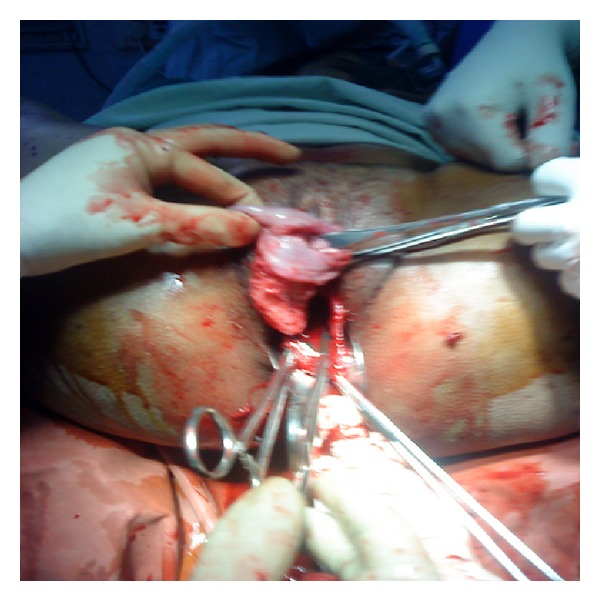
Completely avulsed cervix from lower uterine segment.

 A healthy 25-years-old, G_5_P_2_A_2_ was referred to our hospital for postabortal bleeding with retained placenta. She gave history of expulsion of fetus 15 minutes prior, following a second trimester termination of pregnancy with intravaginal misoprostol of 3 doses, sixth hourly, as told by the patient, the exact dose was not known. Her obstetric history revealed that she had two fullterm home deliveries, two second trimester terminations of pregnancy: one for missed abortion and another for anomalous fetus. Her last abortion was one and a half years back. On examination, the patient was pale, sweating and air hungered (PR-110 bpm, BP-90/50 mmHg). Per abdomen examination showed that uterus was well contracted and retracted and was about 16 week size. Vulval examination showed bleeding and three tampons were removed from the vagina. The placenta and membranes were removed from the vagina, which were already separated. Then the patient started bleeding profusely. On further examination, the cervix which was completely avulsed from lower uterine segment (Figure 1) and attached only anteriorly to the vault was seen. External os was admitting only tip of finger. Lower end of the uterus could not be identified. Depending on the examination findings, uterine rupture with complete cervical avulsion was diagnosed, and it was decided to go for laparotomy.

 At laparotomy, uterus was well contracted and retracted and the bladder was intact. There was a broad ligament haematoma, extending up to lateral pelvic wall on either sides, and also a haematoma was seen behind the uterovesical fold. We decided to do bilateral internal iliac artery ligation first, as there was profuse bleeding from the lower end of the injured uterus. Since repair of completely avulsed cervix to lower end of uterus was difficult and she already had two children, it was decided to go ahead with hysterectomy.

At hysterectomy, lower lip of both anterior and posterior wall of uterus was difficult to identify. Avulsed cervix attached only anteriorly to vault was removed. Continuous oozing was present around the vault. Vault was closed with continuous locking sutures. Hemostasis was achieved as much as possible, gel foam was placed and pelvic peritonization was done. Two units of blood transfusions were given intraoperatively and one point postoperatively. Her haemoglobin which was, 11.6 gm% at admission, had become 6.7 gm% on first postoperative day. Sutures were removed on the eighth post-op day and discharged on the 10th day.

## 3. Discussion

 There is gradual a increase in second trimester pregnancy termination due to prenatal screening for diagnosis of severe fetal cardiovascular and skeletal malformations [2]. In such cases, examination of the fetus after medical abortion gives us valuable informations, like to confirm the congenital anomaly and evaluate for subsequent risks of recurrence and thus helps us to counsel the patient.

 Maternal morbidity and mortality following induced abortions are directly proportional to the gestational age at termination. They increase with increasing gestational age [5]. Uterine rupture and hysterectomy are uncommon, but are inevitable complications of any method of second trimester pregnancy termination [2]. These are more frequent with intravaginal misoprostol which is the most preferred method. There is no fixed dosage, protocol/schedule, or route of administration for use of misoprostol for second trimester MTP. All studies have used a different regimen.

There is also no fixed dosage schedule as per the FOGSI (Federation of Obstetrics and Gynecology of India) guidelines [6] for the use of misoprostol in second trimester MTP. WHO and RCOG [7] recommend 800 *μ*g of misoprostol for induction followed by 400 *μ*g every third hourly for the maximum of 4 doses. They also recommend cervical priming with mifepristone 200 *μ*g, 36–48 hours before induction. Total dose recommended by these both organizations is 2400 *μ*g per day. In a study by Tayade et al. [3], there were no complications in 120 cases when the total dose used was <1000 *μ*g in 24 hours.

 There is a case report of spontaneous uterine rupture in second trimester, in an unscarred uterus due to placenta percreta [8]. So any intervention for induced abortion, especially with intravaginal misoprostol, increases the chances of rupture, more so in the second trimester termination of pregnancy.

 Misoprostol has replaced all other methods for pregnancy termination because of its higher success rate due to its specific myometrial sensitivity [2] as well as it is cheap, stable at room temperature, can be stored for long time, and easily available. In addition, it has fewer gastrointestinal side effects and limited effect on bronchi and blood vessels [9].

 After oral administration, plasma concentration of misoprostol reaches peak after approximately 30 minutes and clears off rapidly with a terminal half life of 20–40 minutes, whereas after vaginal administration, the level increases gradually reaching maximum after 70–80 minutes but remains for significantly longer time [2]. Pharmacokinetic studies have shown that systemic bioavailability of vaginal misoprostol is longer than orally administered misoprostol.

 Incidence of rupture uterus during second trimester pregnancy termination was 0.28% in women with previous caesarean section and 0.004% in women without prior caesarean, according to a systematic review of 16 studies by Goyal [2]. This risk further increases if oxytocin is used along with PGE1 [10]. In a retrospective study by Chapman et al. the risk of uterine rupture was 3.8% with PGE1 and oxytocin in women with a prior caesarean delivery [10]. In a low-profile setup this can result in significant mortality and morbidity, especially if domiciliary treatment is adopted.

 MTP is legalized, but sex determination MTP is prohibited in most of the countries. But second trimester termination, after sex determination is continuing and is being done by unqualified people, especially in countries like India. This further increases the risks of maternal morbidity and mortality.

 Hence, before second trimester pregnancy termination, the patient should be counseled about the complications, and obstetricians should be conscious of the rare but inevitable complications, so that they can be diagnosed and treated early, to decrease the morbidity and mortality. Specified dosages and protocols should be used for limited time and alternative methods employed if it fails. Chances of recurrence of rupture in next pregnancy will be high that is, 4%–19%. So, either tubal ligation should be done or advice elective caesarean section in next pregnancy [11].

## 4. Conclusion

 In the present days, there is a gradual increase in second trimester pregnancy termination, due to prenatal screening for diagnosis of severe fetal cardiovascular and skeletal malformations and also due to social reasons. Misoprostol which is the commonly used method for second trimester termination of pregnancy should be used cautiously, as life-threatening complications are associated with this. If it fails, alternative methods should be used. Patient should be counseled about the complications, and obstetricians should be aware about the uncommon but inevitable complications like uterine rupture, postabortal bleeding needing hysterotomy and blood transfusions. Also the second trimester terminations of pregnancy should be done in a place where facilities for dealing with the above complications are available.
